# Predicting Depression, Anxiety, and Stress Levels from Videos Using the Facial Action Coding System

**DOI:** 10.3390/s19173693

**Published:** 2019-08-25

**Authors:** Mihai Gavrilescu, Nicolae Vizireanu

**Affiliations:** Department of Telecommunications, Faculty of Electronics, Telecommunications and Information Technology, University “Politehnica”, Bucharest 061071, Romania

**Keywords:** affective computing, stress prediction, depression prediction, anxiety prediction, neural networks, face analysis

## Abstract

We present the first study in the literature that has aimed to determine Depression Anxiety Stress Scale (DASS) levels by analyzing facial expressions using Facial Action Coding System (FACS) by means of a unique noninvasive architecture on three layers designed to offer high accuracy and fast convergence: in the first layer, Active Appearance Models (AAM) and a set of multiclass Support Vector Machines (SVM) are used for Action Unit (AU) classification; in the second layer, a matrix is built containing the AUs’ intensity levels; and in the third layer, an optimal feedforward neural network (FFNN) analyzes the matrix from the second layer in a pattern recognition task, predicting the DASS levels. We obtained 87.2% accuracy for depression, 77.9% for anxiety, and 90.2% for stress. The average prediction time was 64 s, and the architecture could be used in real time, allowing health practitioners to evaluate the evolution of DASS levels over time. The architecture could discriminate with 93% accuracy between healthy subjects and those affected by Major Depressive Disorder (MDD) or Post-traumatic Stress Disorder (PTSD), and 85% for Generalized Anxiety Disorder (GAD). For the first time in the literature, we determined a set of correlations between DASS, induced emotions, and FACS, which led to an increase in accuracy of 5%. When tested on AVEC 2014 and ANUStressDB, the method offered 5% higher accuracy, sensitivity, and specificity compared to other state-of-the-art methods.

## 1. Introduction

Stress, depression, and anxiety are emotional states that affect our mental and physical health. Research has shown that these three emotional states are strongly connected: chronic stress can activate defensive mechanisms in the brain that lead to anxiety; constant anxious feelings can cause depression; and, subsequently, depressive states intensify mental and physical stress [1]. This connection indicates that evaluating the three emotional states together would be more suitable than analyzing them separately. Knowing the severity of stress, depression, or anxiety associated with a subject as well as monitoring patterns over time can help in acting proactively to stop chronic mental problems (such as major depressive disorder (MDD), generalized anxiety disorder (GAD), or post-traumatic stress disorder (PTSD)) from developing and assist affected individuals in their day-to-day activities.

In this research, we propose a nonintrusive architecture for determining depression, anxiety, and stress levels by analyzing the facial dynamics of individuals using the Facial Action Coding System (FACS). We, therefore, aimed to build a low-cost nonintrusive system that provides high accuracy and that can be used by health practitioners for assessing and monitoring the severity of the three emotional states, and it also has the advantage of functioning in real time (and allowing experts to analyze severity patterns over time) as well as being less subjective than questionnaire-based methods (which are known to be prone to bias, such that different psychologists could provide different results [2]). We also describe the first research in the literature that studied the relationship between FACS and the Depression Anxiety and Stress Scale (DASS), a commonly used tool for evaluating the three emotional states. We show the results obtained from testing the proposed architecture using intrasubject and intersubject methodologies, and we compared our system in terms of accuracy, sensitivity, and specificity to other state-of-the-art methods used for similar tasks. Apart from being the first study in the literature that has analyzed the relationship between FACS and DASS, this study also proposes a novel architecture that is based on three layers, with the aim of providing DASS levels with high accuracy and with a low prediction time: in the first layer, a method based on Active Appearance Models (AAM) and set of multiclass Support Vector Machine (SVM) classifiers are used for fast Action Unit (AU) classification; in the second layer, a matrix is built containing the intensity levels of the classified AUs; and in the third layer, an optimal feedforward neural network (FFNN) analyzes the matrix from the second layer as a pattern recognition task, predicting the stress, anxiety, and depression levels. The proposed architecture can not only act as a tool for health practitioners, allowing them to evaluate the severity of the three emotional states, but can also be used for a wide range of other applications, including virtual psychology [3], computer-driven therapy [3], personalized health assistance [3], career counseling [3], and even diagnoses of physical diseases that have different stress, anxiety, or depression patterns as symptoms [4,5,6]. 

There are multiple ways of assessing levels of stress, depression, and anxiety, which can be divided into intrusive or contact-based methods and nonintrusive noncontact approaches. The intrusive or contact-based approaches refer to using specific sensors for measuring different parameters such as heart rate variability (HRV) [7], electrodermal activity [8], or an electroencephalogram (EEG) [9,10,11]. They have the advantage of being more precise, but are considerably more costly and need more effort from health practitioners and patients. On the other hand, nonintrusive noncontact approaches such as facial or speech analysis or questionnaire-based methods are easier to use and are less expensive, but are usually less accurate. In the next paragraph, we focus on detailing the current nonintrusive noncontact methods based on facial analysis used for predicting the three emotional states.

For evaluating stress, a significant study was conducted by N. Sharma et al. [12] in which a video database for a temporal thermal spectrum (TS) and visible spectrum (VS) called ANUStressDB was proposed. Researchers used a model based on local binary patterns on three orthogonal planes (LBP-TOPs) for stress detection, which was designed to evaluate histogram dynamic thermal patterns (HDTPs) and exploit the spatiotemporal features from videos by using an SVM-based classifier that classifies the subject as stressed or not. The proposed system offers an accuracy of 86%. This research was extended by R. Iran et al. [13] by extracting facial features from superpixels and providing them as input to different types of classifiers that discriminate between stressed and nonstressed subjects. The method was tested on the same ANUStressDB [12] and offers 3% higher accuracy compared to the method based on LBP-TOPs [12]. Another method was presented in Reference [14], with researchers assuming that mouth activity contains enough relevant information to evaluate stress. They proposed a semiautomated algorithm based on Eigen features and used template matching to classify mouth activity related to mouth openings and mouth deformations. Over 89% accuracy is obtained for discriminating between stressed and nonstressed subjects. Prasetio et al. [15] proposed a method for stress classification (neutral, low stress, high stress) by analyzing images with frontal faces divided into three parts: eyes, nose, and mouth. The facial features are extracted using difference of Gaussians (DoG), histogram of oriented gradients (HOGs), and discrete wavelet transform (DWT) methods. The histogram features determined are processed by a convolutional neural network (CNN) used to model the facial stress expressions. Tested on a facial recognition technology (FERET) [16] database using five-fold cross-validation, the system offers over 82% accuracy for stress classification.

For evaluating depression, local curvelet patterns in three orthogonal planes (LCBP-TOPs) have been successfully used for analyzing facial features and determining whether a subject is affected by depression or not, with over 73% accuracy [17]. Similarly, LBP-TOPs have been used [18] to analyze a set of visual cues in temporal segments using Fisher vectors (FVs). The method was tested on the Audio-Visual Emotion Challenge (AVEC2014) German database [19] and provided 75% accuracy in distinguishing between depressed and nondepressed subjects. LBP-TOPs have also been used for computing a temporal visual words dictionary [20] that was fetched to an SVM-based classifier to determine whether the subject was affected by depression or not, reaching an accuracy of 77%. The multiscale entropy (MSE) method has also been used [21] for the quantization of the temporal variability of facial expressions extracted from unsupervised features in video recordings collected from patients affected by MDD. Researchers have used a dynamic latent variable model (DLVM) for learning, and the results showed that unsupervised learned features could be used to distinguish between different levels of depression, with an accuracy of over 70%. Yang et al. [22] proposed an audio-visual multimodal depression recognition framework based on deep convolutional neural networks (DCNNs) and deep neural networks (DNNs). Each modality is provided as input to a DCNN that learns high-level global features. These features are then provided to a DNN to predict the Patient Health Questionnaire (PHQ)-8 depression scores. The PHQ-8 scores are then processed by a DNN for final prediction. Tested on the AVEC2014 database [19], the system offers an accuracy of over 85% for depression prediction from speech and face modalities. Similarly, De Melo et al. [23] fused multiple 3D CNNs (C3D) with the purpose of improving the accuracy of standard CNN-based methods for predicting depressive behaviors. The proposed method proved to be fast and reduced the number of model parameters and the risk of overfitting the networks, offering accuracy of over 80% for depression prediction when tested on the AVEC2014 database [19]. Zhu et al. [24] presented a method that uses joint tuning layers to integrate facial appearances and dynamics through a set of DNNs. Tested on AVEC2014 [19], the method could predict with over 80% accuracy depressive behavior. Yang et al. [25] proposed a system for hybridizing deep and shallow models for depression prediction from audio, video, and text descriptors. Researchers employed a DCNN–DNN model for audio-visual multimodal depression recognition using the PHQ-8 framework, and a paragraph vector (PV) analyzed the interview transcript using an SVM-based model in order to infer the physical and mental conditions of the subject. A random forest (RF) model has been used for depression classification from estimated PHQ-8 scores and inferred conditions of the subject. Experimental results showed that the proposed hybrid method improved the accuracy for depression prediction to over 74% when tested on the AVEC2014 database [19]. Deep regression networks (DepressNets) have also been used for predicting depressive disorders from facial features [26]. A DCNN with a global average pooling layer was first trained on faces affected by depression, which allowed the identification of salient regions of the input image related to different depression severity scores. Further, a multiregion DepressNet was used that contained multiple local deep regression models for different face regions that were jointly learned, and their responses were fused. The method was tested on the AVEC2014 database [19], offering over 82% depression prediction accuracy.

Evaluating anxiety is done separately or together with stress assessment. A way to automatically predict anxiety scores using the different facial expression classification scores was proposed in Reference [27]. Researchers determined a set of features that were analyzed in both raw data and principal component analysis (PCA)-transformed space and looked for correlations between facial expressions and anxiety levels. The results showed that the approach offers reasonable scores for anxiety prediction and confirmed the relationship between facial expressions and anxiety. Active shape models (ASMs) have also been used [28] to extract 68 feature points from five facial areas that were fetched to an SVM-based classifier in order to assess if 18 postgraduates suffered from public speaking anxiety (PSA) in their master’s thesis defense. An accuracy of 99% was obtained when comparing the results provided by the ASM-based method to those from self- and audience assessments. Pediaditis et al. [29] started from a similar premise as the current paper, mentioning the shortcomings of questionnaire-based methods that are commonly used to evaluate stress and anxiety, knowing that these are subjective and prone to bias. They studied the facial features that could be used to predict stress or anxiety efficiently. By combining different classification methods and analyzing sets of 9 to 10 features from different facial areas targeted separately, the methods offered prediction accuracy for both stress and anxiety of up to 73%.

In terms of FACS, limited research has been conducted that used this model for predicting depression, anxiety, or stress. McIntyre et al. [30] used action unit (AU) groups called region units (RUs) determined based on active appearance models (AAMs) and employed Multiboost to classify the facial expressions. The results showed that using this method, depression could be predicted with up to 75% accuracy. Researchers in Reference [31] presented another FACS-based method to investigate the relationship between symptoms of depression and facial expressions over time. Several subjects under depression treatment were recorded during a series of clinical interviews, and their facial expressions were analyzed using FACS. The study showed that when depression symptoms were high, the subjects displayed more facial expressions associated with emotions such as contempt and sadness, confirming the social risk hypothesis of depression [31], which states that people suffering from severe depression withdraw from any social activity and enter a self-protective state. As the symptoms become less severe, subjects show more openness to socializing, and emotions such as happiness or surprise become more conspicuous. Of note is that there have been several studies that have used Red Green Blue—Depth (RGB-D) databases for facial expression recognition, which typically can be collected by means of a Microsoft Kinect sensor with low computational costs, allowing for fast convergence. Szwoch [32] proposed a Facial Expression and Emotion Database (FEEDB) by taking advantage of the fast acquisition, indexing, and storing of the KinectRecorder tool, significantly reducing the time usually spent on data acquisition, indexing, and validation when creating a fully indexed database. In Reference [33], the database was used to predict nine emotions through an algorithm that used local movement detection within the area of the face in order to recognize actual facial expressions. The system offered an average recognition accuracy of above 50%, with the main advantage that it was highly independent of lighting conditions and could provide high accuracy when the sensor was very close to the user. Similarly, Ballihi et al. [34] used upper body movements and facial expressions analyzed from a dataset containing RGB-D movies, extracting a set of positive and negative cues from 2D (RGB) information. A geometric method was used to model depth flows and determine human body dynamics from a depth dataset. Given the temporal changes in pixel and depth intensity, depth features were employed to describe the relationship between the changes in the upper body movements and the affect. The experiments, conducted on a Cam3D dataset [35], showed promising results in detecting people’s moods using upper body postures and monitoring their evolution in time. An interesting study using RGB-D sensors (color and depth) was one conducted by Jaiswal et al. [36], who proposed a framework for helping with the diagnosis of attention deficit hyperactivity disorder (ADHD) and autism spectrum disorder (ASD), neurodevelopmental conditions that impact a significant population of children and adults. Researchers focused on detecting several behavioral markers, providing a fully automatic end-to-end system to predict ADHD and ASD in adults. They performed facial expression analysis based on dynamic deep learning (DDL) and 3D analysis, obtaining a classification rate of 96% for controls versus condition (ADHD/ASD) groups and 94% for the comorbid (ADHD and ASD) versus ASD-only group.

With all this research previously conducted as a premise, this paper introduces for the first time in the literature a neural network-based architecture for predicting levels of depression, anxiety, and stress based on FACS in a nonintrusive and real-time manner. We have also conducted the first study in the literature that has analyzed the correlations between FACS AUs and DASS levels.

## 2. Materials and Methods

### 2.1. Psychological Grounds

This research relied on two popular tools, which we describe in the next paragraphs: the Depression Anxiety Stress Scale (DASS) [37] and the Facial Action Coding System (FACS) [38].

DASS is a tool broadly used by psychiatrists to determine the severity of three emotional states: depression, anxiety, and stress. In this study, we employed a DASS version with 42 items, which uses a specific self-analysis questionnaire (SAQ) in which subjects are asked to self-report on 14 items associated with emotional states, as follows [37]:

- Depression. Dysphoria, anhedonia, inertia, hopelessness, devaluation of life, lack of interest, self-deprecation;
- Anxiety. Autonomic arousal, situational anxiety, skeletal muscle effects, subjective experience of anxious affect;
- Stress. Difficulty relaxing, nervous arousal, easily agitated/upset.

For each of these items, the subjects answer particular questions by self-rating, using a score on a scale from 0 to 3. The sum of all scores obtained from each item determines the severity of the emotional state as follows: Normal (0–9 score for depression, 0–7 score for anxiety, 0–14 score for stress), Mild (10–13 score for depression, 8–9 score for anxiety, 15–18 score for stress), Moderate (14–20 score for depression, 10–14 score for anxiety, 19–25 score for stress), Severe (21–27 score for depression, 15–19 score for anxiety, 26–33 score for stress), and Extremely Severe (28+ score for depression, 20+ score for anxiety, 34+ score for stress).

FACS is a system developed by Eckman and Friesen [38] for analyzing the microexpressions on human faces and determining the emotion of the analyzed subjects. It is based on the idea that each emotion can be associated with different facial muscle patterns and that by analyzing facial regions where these muscles are activated, we can determine the emotion of the individual. The main advantage of FACS compared to other face analysis methods is that it can determine hidden emotions, even if the subject is trying to mimic different ones. FACS divides the face into 46 action units (AUs), which can be either nonadditive (the activity of an AU is independent of the activity of another AU) or additive (when an AU is activated, it triggers the activation of another AU or group of AUs). Based on FACS, the AUs’ presence and intensities are measured using the following levels: Level O, AU is not active; Level A, trace; Level B, slight; Level C, marked or pronounced; Level D, severe or extreme; and Level E, maximum.

FACS is typically used to determine the emotions of individuals. In this paper, we took this challenge further and tried to determine the possibility of using FACS to evaluate the severity of depression, anxiety, and stress. Although limited, there is previous research that has suggested possible connections between emotions and emotional states (depression, anxiety, or stress). In Reference [39], researchers proposed an emotions recognition system for classifying four basic emotions (happiness, fury, sadness, and neutral) using a speech modality, and they showed that there were links between these four emotions and depression or anxiety. Similarly, in Reference [40], researchers studied emotion-related variables in correlation with anxiety and depression, and they showed that there was a close link between depression and a high frequency of negative emotions and a low frequency of positive emotions, as well as between anxiety and specific patterns in the frequency of the emotional experience. Similarly, stress was linked to increased negative emotions, such as anger [41], while positive emotions (such as happiness) were linked to an increased physiological ability to recover from chronic stress [42].

In the following section, we present how these two tools are used in our proposed architecture as well as detail its structure, the neural network configuration, and the training and testing phases. 

### 2.2. Proposed Architecture

We propose a novel nonintrusive multilayer neural network-based architecture for predicting DASS levels by analyzing facial expressions using FACS. The architecture has a unique structure on three layers: in the first layer, the video frames are normalized, and the analyzed AUs are classified using an AAM-based method and a set of multiclass SVMs; in the second layer, a matrix is built based on the intensity levels of the selected AUs; and in the third layer, a neural network-based structure is developed that analyzes the patterns from the matrix built in the second layer and predicts the DASS levels (Normal, Mild, Moderate, Severe, or Extremely Severe) for each of the three emotional states. The reason for building a system on three layers instead of an end-to-end learning model was that we wanted to study the possible correlations between FACS and the analyzed AUs (classified in the first layer) and the predicted DASS levels (from the top layer). The study is also part of a broader research project aiming to build a system for diagnosing mental disorders and physical diseases for applications in the healthcare industry in English-speaking countries by analyzing information collected from different sources in a noninvasive manner. Hence, we opted for a modular approach that would allow us to easily add other modalities as input, other psychological conditions as output, as well as weight and fuse different modalities in the second layer matrix, reducing these problems to pattern recognition tasks.

The architecture was built using a Java virtual machine (JVM) with Eclipse as an integrated development environment (IDE) and Scala as a programming language using Spark Library. The testbed has an Intel i7 processor with 8 GB of random access memory (RAM) running Linux Solaris 11.3 as an operating system (OS). In the following subsections, we detail the three constituting layers.

#### 2.2.1. The First Layer

As mentioned previously, the last FACS revision proposed 46 primary AUs [43], but only 30 of them were anatomically correlated with facial muscle contraction: 12 in the upper part of the face and 18 in the lower part [43]. Out of these 30 AUs, we analyzed only the following:

- Upper face: AU2 (outer brow raiser), AU4 (brow lowerer), AU7 (lid tightener), AU43 (eyes closed), AU6 (cheek raiser), AU1 (inner brow raiser), AU5 (upper lid raiser), and AU45 (blink);
- Lower face: AU8 (lips toward each other), AU25 (lips part), AU16 (lower lip depressor), AU11 (nasolabial deepener), AU13 (sharp lip puller), AU15 (lip corner depressor), AU17 (chin raiser), AU10 (upper lip raiser), AU18 (lip pucker), AU19 (tongue show), AU20 (lip stretcher), AU22 (lip funneler), AU23 (lip tightener), AU14 (dimpler), AU12 (lip corner puller), AU24 (lip pressor), AU26 (jaw drop), AU27 (mouth stretch), AU9 (nose wrinkler), and AU28 (lip suck).

All of these selected AUs are part of the standard set used in most facial expression recognition (FER) systems based on FACS [44,45]. We excluded AU41 (lid droop), AU42 (slit), and AU46 (wink) from the upper part of the face because these AUs were not coded with intensity levels in the last FACS revision [43], and a binary classification (AU present/absent) in a context where all other AUs were evaluated based on a level from O to E could negatively impact the accuracy of the system. Apart from the 28 AUs mentioned above, we also analyzed AU33 (cheek blow), AU34(cheek puff), and AU35 (cheek suck) to enrich the facial features of the cheek areas, as these 3 AUs were coded with intensity levels in the latest FACS revision [43]. Therefore, we used 31 AUs that could be classified fast and with high accuracy: For the remaining AUs, more complex methods would have been required, which would also have required more processing time, so they were not evaluated in this study, since we aimed to build a system able to predict the DASS levels in real time. Another reason for which we selected this set of AUs was that they are present in several large databases employed for testing FER systems [44,45], such as Cohn-Kanade extended (CK+) and MMI, which we used in this study for evaluating the accuracy of the AU classification task. All AUs analyzed were treated individually, as nonadditive.

The first step is face detection, for which we created a statistical model of the skin, and we classified pixels from the analyzed frame in facial and nonfacial pixels, obtaining an image containing only the areas classified as related to the face [46]. We also used a noise reduction filter based on Gabor filters and non-negative matrix factorization [47]. After these filters were applied, we obtained a candidate face for the current video frame. We continued with determining the position of the eyes, as they are known to have the lowest variation of all face elements [48]. Therefore, after face detection, we evaluated the image horizontally, and we calculated the number of white pixels in each row. The row that contained the highest number of white pixels from the upper part of the candidate face was considered the median of the block containing the eyes, while the one from the lower part of the candidate face was the median of the mouth block. Because we know that the eyes are located between 2/20 and 9/20 of face height and that the mouth is located between 11/20 and 15/20 of face height [49], we performed additional validations based on these ratios to ensure that the blocks delimited as pertaining to the eyes and mouth were accurate. This method proved to be up to 80% faster than other state-of-the-art methods based on facial landmarks when analyzing frontal faces. Therefore, we opted for this method, because in our study, we wanted to exclusively analyze frontal face video recordings and because we aimed for a real-time prediction of the three emotional states, which requires fast convergent methods.

**Figure 1:**
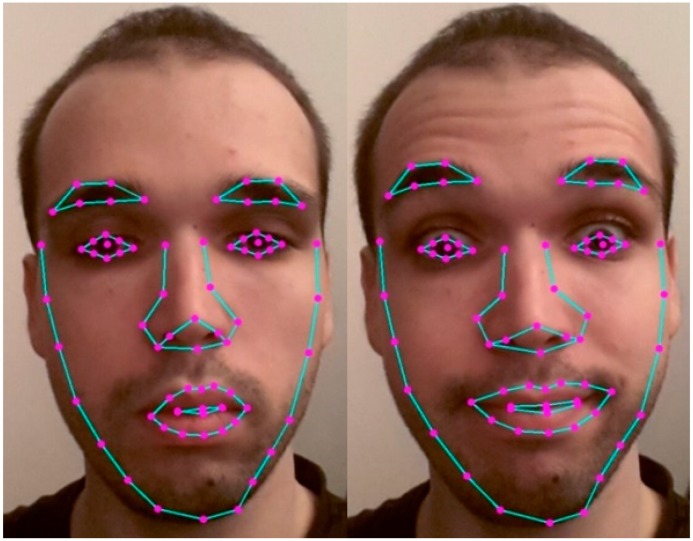
Facial landmarks for AAM algorithm and different facial expressions.

After the face and the eyes and mouth blocks were detected, we extracted facial features using AAM [50], as this method is fast for similar problems [51,52] and offers good results for AU detection [53]. We employed a Principal Component Analysis (PCA) to produce a parameterized model describing the faces used for training and to estimate new faces [50,51]. Matching the model’s parameters with the candidate faces was done in the first instance by matching the eye and mouth areas, then using a steepest descent algorithm and Jacobian matrices to match the entire parametrized model [51]. The parameters of the model were evaluated iteratively until they provided the most accurate description of the candidate face. We used a vector of coordinates from a set of facial landmarks called shape [51]. All of the shapes were aligned with the standard model, and we studied the shape variation using generalized Procrustes analysis (GPA) [54]. We determined a set of nonrigid parameters for 72 facial landmarks (as seen in Figure 1), and testing the algorithm on 128 different subjects using leave-one-out cross-validation, we determined, through trial and error, that 1500 iterations were required to determine these parameters with over 90% accuracy. 

**Figure 2:**
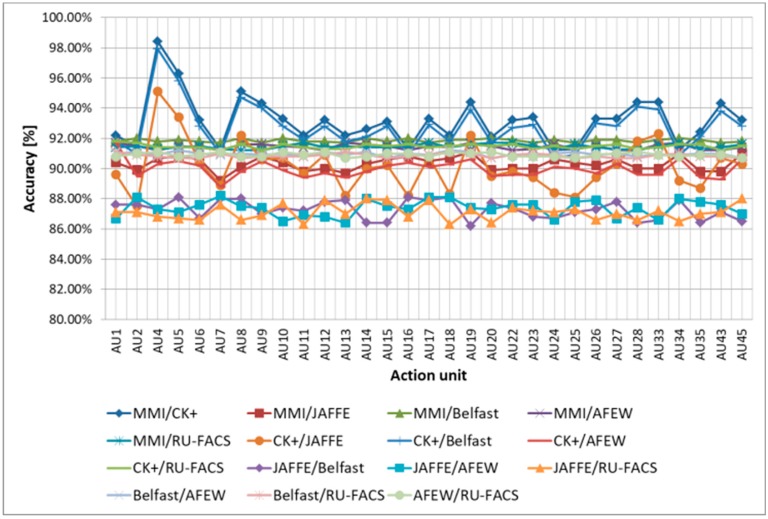
AU classification accuracy for cross-database tests.

The 72 nonrigid parameters are provided as input to a set of 31 multiclass SVMs used for the classification of the 31 AUs’ intensity levels, with one multiclass SVM for each analyzed AU. We used SVMs for classification because they have proven to be accurate and fast for similar problems [55]. The SVM classifiers search for the hyperplanes that maximize the margins of a class and use a vector to separate hyperplanes and a bias estimated to minimize the error of the training set. We used LIBSVM [56] for training and testing the multiclass SVM classifiers. Each such classifier receives as input the 72 nonrigid parameters computed for a frame from the video sample and is trained to associate the analyzed frame with 1 of 6 classes (for the 6 AU intensity levels). The output of the SVMs will, therefore, provide the intensity level of the analyzed AU (O, A, B, C, D, or E). The multiclass SVMs were evaluated in cross-database tests on the CK+ [57], MMI [58], Japanese Female Facial Expression (JAFFE) [59], Belfast [60], Acted Facial Expressions in the Wild (AFEW) [61], and RU-FACS [62] databases. The results obtained are presented in Figure 2, where we can observe the classification accuracy for each AU in each of these cross-database tests. The results were averaged such that, for example, for MMI/CK+, we trained the system on MMI and tested it on each sample from CK+, and then we trained on CK+ and tested each sample from MMI, averaging the accuracy of all tests conducted. 

**Figure 3:**
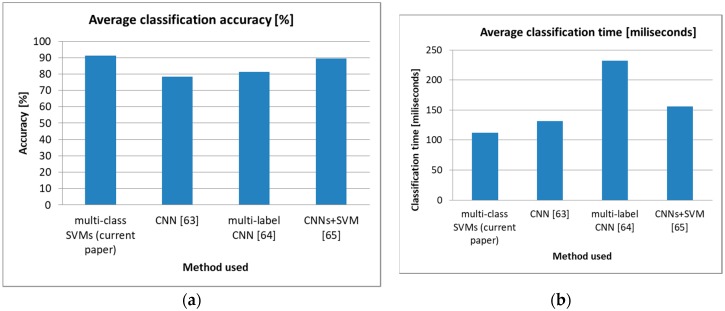
Comparison of state-of-the-art methods for AU classification. (**a**) Average classification accuracy [%], (**b**) Average classification time [miliseconds].

As can be observed, for most of the cross-database tests, all AUs were classified with over 90% accuracy, which offered a strong baseline for the more complex task of predicting the DASS levels. We also observed a lower accuracy obtained when the JAFFE [59] database was used for training or testing, mainly because this database contains Mongoloid subjects, while the other databases have Caucasian subjects. Given the fact that in our database, we used Caucasian subjects exclusively, this did not pose a concern for further tests conducted to evaluate DASS levels. We compared the average accuracy obtained by using this multiclass SVM-based method to other state-of-the-art CNN-based methods for AU classification (CNN-based classifiers [63], multilabel CNN classifiers [64], and a combination of CNN-based and SVM-based classifiers [65]) that are known to offer high classification accuracy, and the results are displayed in Figure 3. As can be observed, the proposed multiclass SVM-based method offered an accuracy comparable to the one obtained using the method with CNN-based and SVM-based classifiers proposed in Reference [65], but it was significantly faster (hence why we used it in this study, as we aimed to build a system able to predict DASS levels in real time).

#### 2.2.2. The Second Layer

**Figure 4:**
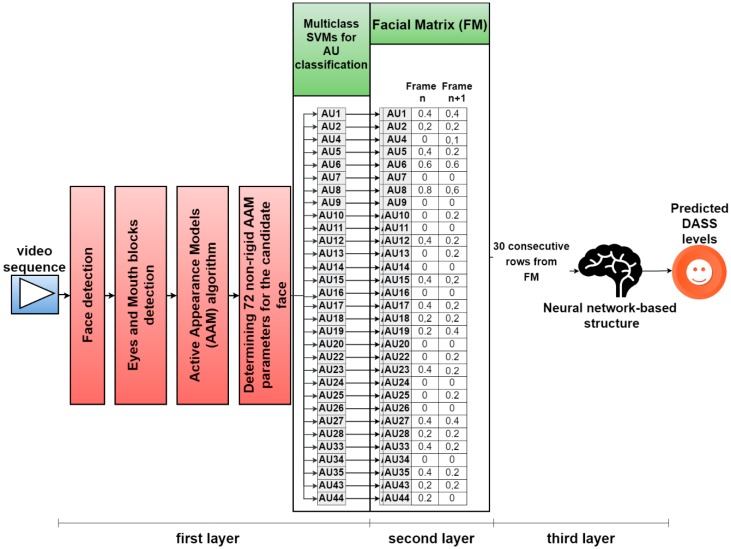
Overall architecture for predicting DASS levels by analyzing facial features using FACS.

The output of the first layer consists of an intensity level from O to E estimated for each of the 31 AUs. This level is normalized in [0,1] intervals as follows: level A = 0.2, level B = 0.4, level C = 0.6, level D = 0.8, level E = 0.9, AU absence = 0. This normalization is required to allow the neural network-based pattern analysis in the third layer. With the normalized values, in this second layer, a facial matrix (FM) is built. The FM contains a column for each frame of the analyzed recording, each column consisting of the intensity levels of all the AUs present in that given frame. Figure 4 shows the structure of the first and second layers. When 30 new columns are added to the FM (corresponding to 30 frames), they are sent to the third layer, where a neural structure analyzes the patterns and provides the predicted DASS levels of the analyzed subject.

#### 2.2.3. The Third Layer

As mentioned in previous sections, although there are multiple methods in the literature for predicting the three emotional states, in this paper, we evaluate only neural networks: hence, we aimed to determine the best neural network-based structure and configuration for predicting DASS levels using facial features, with high accuracy. There are typically 4 types of neural networks commonly used: feed-forward neural networks (FFNNs), radial basis function neural networks (RBFNNs), recurrent neural networks (RNNs), and convolutional neural networks (CNNs, a particular type of FFNN). RBFNNs [66] have limited applications in the affective computing (AC) domain and are mainly used for energy restoration systems, while RNNs [67], although designed to process sequential data, have been studied and have proven to be more efficient in speech and handwriting recognition systems, while for facial analysis tasks they have been understudied. On the other side, FFNNs have been successfully used for image processing and voice recognition, offering efficient classification in cases where the input is nonlinear or affected by noise [68]. Based on these aspects, in this paper, we focused exclusively on analyzing how FFNNs can be used to predict DASS levels with high accuracy.

**Figure 5:**
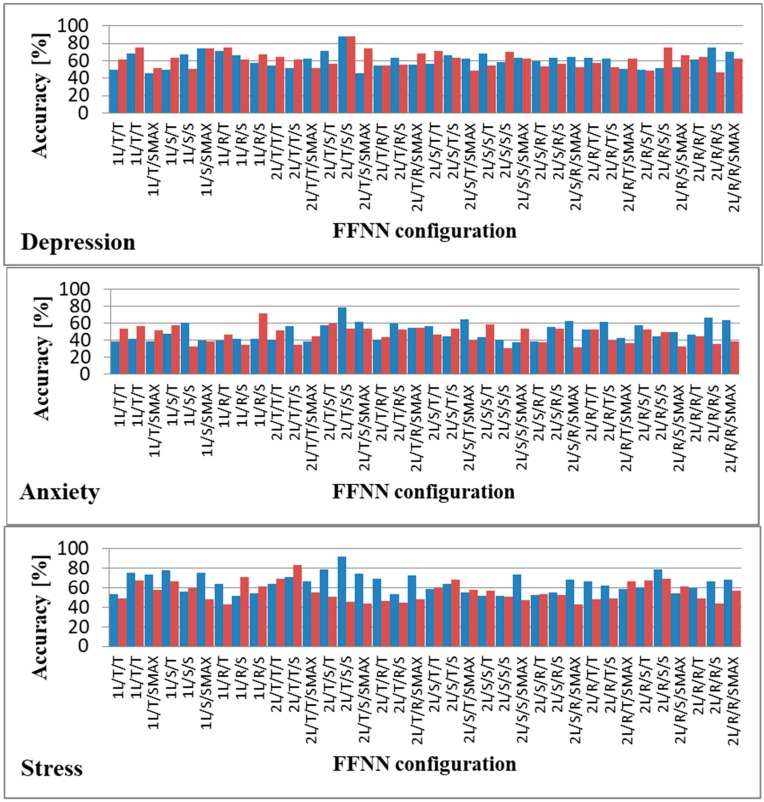
Prediction accuracy using different combinations of neural networks. T: tanh; S: sigmoid; R: ReLU; SMAX: softmax; 1L: one hidden layer; 2L: two hidden layers; **■**: common FFNN for all three emotional states; **■**: dedicated FFNN for each emotional state.

Because for most pattern recognition problems, two hidden layers are enough [69], we only evaluated FFNNs with one or two hidden layers. As activation functions, we evaluated tanh, sigmoid, Rectified Linear Unit (ReLU), and softmax. Because ReLU outputs are in (0, +∞) intervals and given the fact that they are affected by the exceeding gradient problem [70], we only used them in hidden layers. Softmax, on the other hand, has been shown to be inefficient in hidden layers [71], and hence we used it only for the output layer. We also analyzed the use of only one FFNN to predict all three emotional states as well as dedicated FFNNs for each emotional state. Therefore, we evaluated whether a single FFNN can be used to predict depression, anxiety, and stress or whether three FFNNs used for each of the three emotional states provide better accuracy, in each case determining the optimal hyperparameters of the FFNNs. The results obtained after testing all possible combinations are displayed in Figure 5. 

As can be observed, the highest accuracy for the prediction of all three emotional states was obtained when we used a single FFNN to model all three emotional states. This finding shows that depression, anxiety, and stress are strongly connected from a facial point of view, validating the argument mentioned in Reference [1]. The optimal FFNN had 3 output nodes in [0,1] intervals representing the DASS levels predicted for each of the three emotional states, as follows: [0,0.15] for Normal, [0.15,0.3] for Mild, [0.3,0.6] for Moderate, [0.6,0.8] for Severe, and over 0.8 for Extremely Severe. The thresholds for each of the 5 levels were determined as optimal through trial and error. 

**Figure 6:**
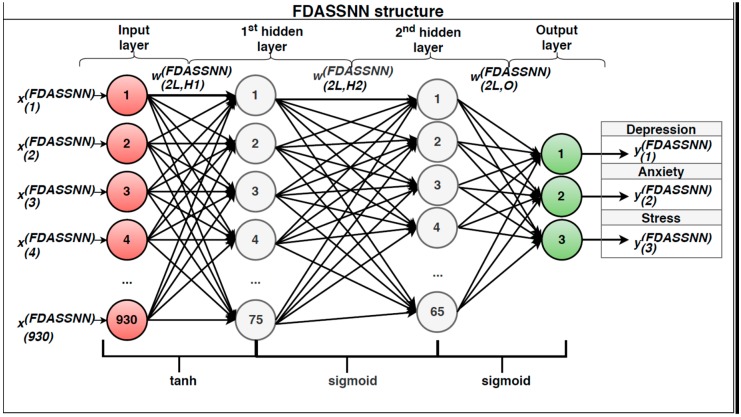
Face Depression Anxiety Stress Scale Neural Network (FDASSNN) configuration and hyperparameters.

We denoted this FFNN: Face Depression Anxiety Stress Scale Neural Network (FDASSNN). The structure determined as optimal had two hidden layers, with tanh as the activation function for the first hidden layer and sigmoid for the second hidden layer and the output layer. We considered $N_{in}$, the number of training vectors provided, as input, with a set of *N*-dimensional input vectors ($X^{\left(FDASSNN\right)}=\left\{x_{\left(n\right)}^{\left(FDASSNN\right)}\right\},\;n=1,2\ldots N_{in}$, such as $x^{\left(\mathrm{FDASSNN}\right)}={\left[x_{\left(1\right)}^{\left(FDASSNN\right)},x_{\left(2\right)}^{\left(FDASSNN\right)},\ldots ,x_{\left(N\right)}^{\left(FDASSNN\right)}\right]}^{T}$); and $K_{out}$, the number of output vectors, with a set of *K*-dimensional output vectors $(Y^{\left(FDASSNN\right)}=\left\{y_{\left(k\right)}^{\left(\mathrm{FDASSNN}\right)}\right\},\;k=1,2\ldots K_{out}$); and we denoted the weight matrix between the input layer and the first hidden layer $W_{\left(2L,H1\right)}^{\left(FDASSNN\right)}$, the weight matrix between the first hidden layer and the second hidden layer $W_{\left(2L,H2\right)}^{\left(FDASSNN\right)}$, the weight matrix between the second hidden layer and the output layer $W_{\left(2L,O\right)}^{\left(FDASSNN\right)}$, $L_{\left(1\right)}^{\left(FDASSNN\right)}$ (the number of hidden nodes from the first hidden layer), and $L_{\left(2\right)}^{\left(FDASSNN\right)}$ (the number of hidden nodes from the second hidden layer). This structure can be observed in Figure 6. 

**Figure 7:**
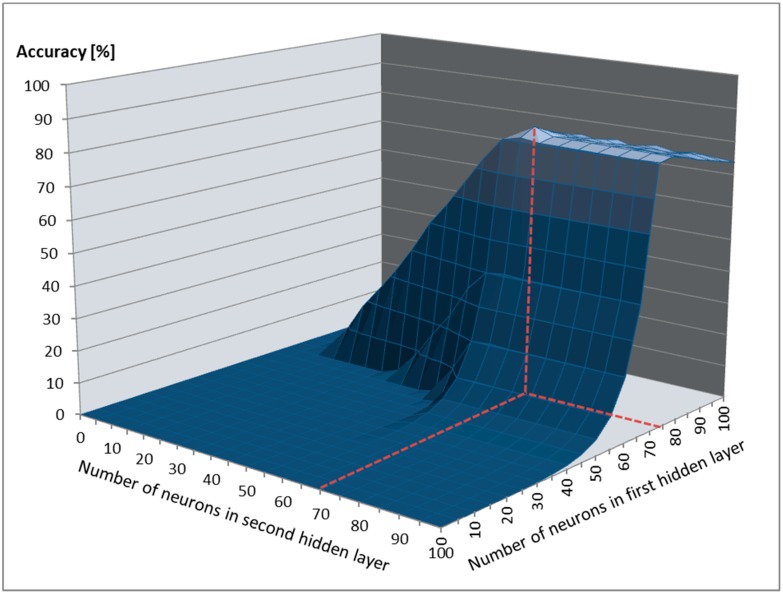
Prediction accuracy based on the number of neurons in hidden layers.

By splitting the training data into a training and validation dataset with an 80:20 dataset split ratio and fine-tuning the FDASSNN hyperparameters, we determined that the optimal number of neurons required in the first hidden layer was 75, with 65 neurons for the second layer (Figure 7). During training, the bias for the hidden layers was set to 0. The optimal learning rate determined was 0.2, the optimal momentum was 0.4, and we required 7000 training epochs to train FDASSNN, with the process lasting 5.5 h. 

As a training method, we used backpropagation because it is largely used for training FFNNs and in pattern recognition tasks, as it is fast and accurate [72]. We used gradient descent (GD) to optimize the weights and biases and minimize the final error, with the purpose of reducing it to below 0.02. We also used the Nguyen–Widrow initialization method [73] to spread the weights in the initial state evenly. The error was calculated using the average absolute relative error (AARE) formula, with $y_{p}$ as desired output and $y_{e}$ as estimated output:

(1) $$\mathrm{AARE}=\frac{1}{N_{in}}\sum_{p=1}^{N_{in}}\left|\left(\frac{y_{p}-y_{e}}{y_{e}}\right)\right|.$$

### 2.3. Operating Modes

The proposed architecture operates in two phases: training and testing.

In the training phase, videos from the training dataset are provided as input to the first layer, frame by frame. In the first layer, the face and the eye and mouth facial blocks are detected, and the 72 nonrigid parameters are determined and provided as input to the set of 31 multiclass SVMs, previously trained on the CK+ database, which output the intensity levels of each of the 31 AUs. The intensity levels are sent to the second layer, which normalizes them in [0;1] intervals and adds the normalized results in a new column to the FM. When 30 new columns are present in the FM, they are sent to the third layer, where the FDASSNN is trained through backpropagation, comparing the predicted output to the expected one (the results obtained from SAQ) and correcting the weights in order to minimize the AARE. We opted for 30 columns because they corresponded to 30 frames, and given the fact that we had a frame rate of 30 fps, they linked to 1 s of facial recording, high enough to catch microexpressions (lasting maximum 500 milliseconds [20]) and low enough to avoid overfitting of the FDASSNN. The training process stops when the AARE is minimized (i.e., has a value lower than 0.02) or when 7000 training epochs are reached. 

In the testing phase, the steps are similar. A video sequence that is not used in the training phase is provided as input to the first layer, frame by frame. The first layer detects the face and corresponding eye and mouth blocks and determines the 72 nonrigid facial parameters, based on which the 31 multiclass SVMs estimate the intensity levels of the 31 AUs. The intensity levels are sent to the second layer, normalized, and added in a new column to the FM. When we have 30 new columns in the FM, they are sent to the third layer, where the FDASSNN outputs the DASS levels. One final rule-based classifier (RBC) is used, which collects the predictions from the FDASSNN and provides a final result, as follows:

- If the same prediction is provided for five consecutive seconds, that prediction is the final output of the system;
- Otherwise, the RBC marks the output as *Undefined,* which means the information is insufficient for a prediction.

**Figure 8:**
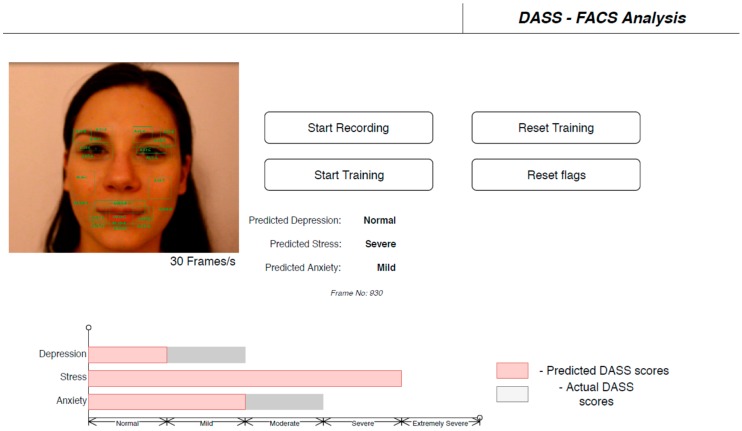
User interface for the application designed to predict DASS levels based on FACS.

The user interface (UI) of the application can be observed in Figure 8.

## 3. Results

In this section, we present the database created for evaluating the proposed architecture, as well as the results obtained from intrasubject and intersubject methodologies. We conclude this section with a comparison between the proposed architecture and other state-of-the-art methods for evaluating depression, anxiety, and stress based on facial analysis.

### 3.1. FACS–DASS Database

**Figure 9:**
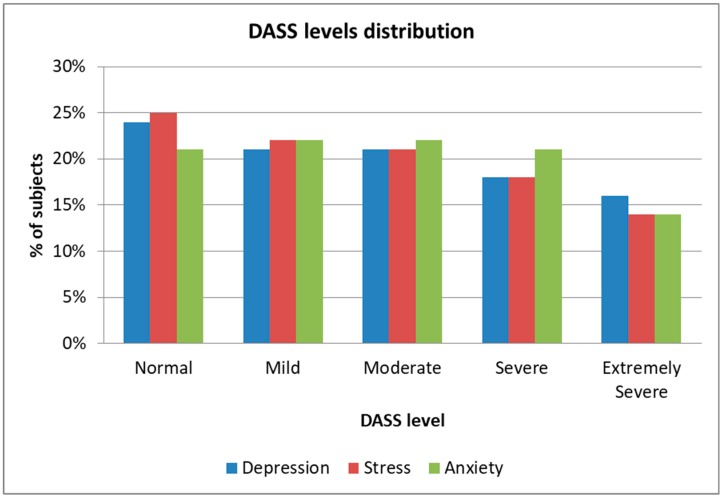
DASS levels distribution.

Because of a lack of a database that links FACS with DASS levels, we created our own database by collecting samples from 128 Caucasian subjects (64 males and 64 females) aged between 18 and 35 years old. The subjects participated in conformity with the Helsinki Ethical Declaration [74] and were chosen in order to display different severities of stress, depression, and anxiety, 20 of them suffering from various stages of MDD, 19 of various stages of GAD, and 17 of PTSD. No subject displayed comorbid conditions (e.g., subjects suffering from MDD and also affected by GAD). The remaining healthy subjects were evaluated by trained psychologists, and they displayed different anxiety, stress, and depression levels, but were not suffering from any specific disorders. The distribution of the DASS levels across the 128 subjects is presented in Figure 9, which shows that we had intersubject variability in order to use the database for testing the proposed architecture with the intersubject methodology.

During each session with each of the 128 subjects, the following samples were collected:

- Frontal face recordings when subjects were asked to watch emotion-inducing videos (inducing the six basic emotions: sadness, fear, happiness, anger, surprise, and disgust). In total, six frontal face one-minute video recordings were collected, one for each emotion;
- Six discrete emotion questionnaire (DEQ) [75] results after the subjects watched each of the six emotion-inducing videos;
- SAQ results after the subjects watched the emotion-inducing videos;
- Five frontal face one-minute video recordings when the subjects were watching emotionally neutral videos;
- SAQ results after the subjects watched emotionally neutral videos.

The subjects were naive participants and were not medicated at the time the samples were collected. Both the SAQ and DEQ results were evaluated by five trained psychologists to ensure they were consistent, and the questionnaire results and associated facial recordings that were not considered proper after reviewing the video recordings and interviewing each subject were excluded (2.5% of the samples were excluded). The DEQ results were used to validate that the emotion induced by the videos was indeed induced in the subject, and recordings where the DEQ provided an emotion other than the one induced were also excluded (2% of recordings were excluded). To induce the six emotions, we used videos from the LIRIS-ACCEDE database [76]. Because these videos were between 8 and 12 seconds, but analyzing facial expressions needed more time, we combined multiple videos for the same emotion in a one-minute compilation. We chose to induce emotions not only to generate emotionally rich samples but also to increase the number of AUs activated in a short time, which was useful when analyzing how the induced emotion influenced predictions as well as the system’s ability to be accurate in naturalistic conditions. For recording, we used a Sony PMW-300 One camera with 30 fps and 1024 × 768 resolution.

The samples were collected for each subject six times, with a break of two weeks between sessions in order to include the possible dynamics of emotional states over time. The average intrasubject DASS variability was 0.8 levels from one session to another, which allowed for further tests using intrasubject methodology. At the end of the three months, we had, for each subject, 36 facial recordings where emotion was induced (one for each emotion from each recording session), 30 facial recordings where no emotion was induced, 36 DEQ results, and 12 SAQ results. Based on how the information was collected, we divided the database into two datasets:

- Dataset (controlled) (DSC), containing samples collected when emotion was induced; and
- Dataset (random) (DSR), containing samples collected when no emotion was induced.

Note that the average time taken to fill in the SAQ was 25.4 min.

To evaluate the proposed system, we used the following statistical measures (where *TP* refers to the number of true positives, *TN* to the number of true negatives, *FP* to the number of false positives, and *FN* to the number of false negatives) [77].

Accuracy (AC):

(2) AC=(TP+TN)/(TP+TN+FP+FN).

Sensitivity or true positive rate (SE/TPR):

(3) TPR=TP/(TP+FN).

Specificity or true negative rate (SP/TNR):

(4) TNR=TN/(TN+FP).

Note that, in both the intrasubject and intersubject methodologies, we evaluated the prediction of each DASS level. For example, when predicting the Normal level, we considered *TP* to be associated with cases where the system correctly predicted the Normal level, *TN* to be associated with cases where the system correctly predicted that it was not a Normal level, *FP* where the system incorrectly predicted the Normal level, and *FN* when the system incorrectly predicted that it is not a Normal level.

### 3.2. Intrasubject Methodology

For intrasubject methodology, we trained and tested the architecture using samples pertaining to the same subject, varying the testing and training datasets (DSC, DSR, or both) and using leave-one-out cross-validation. The results can be observed in Table 1.

**Figure 10:**
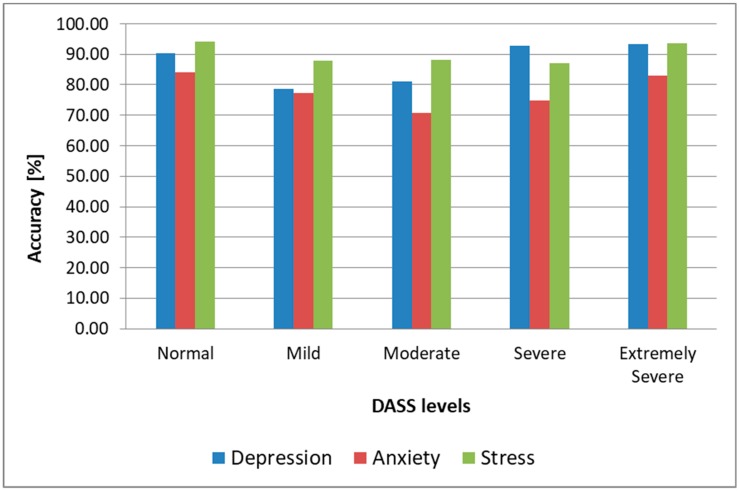
DASS levels prediction accuracy (intrasubject methodology).

Since the output of the architecture is represented by the five DASS levels, a successful prediction was considered to be when the predicted DASS level was the same as the one that resulted from a subject’s completed SAQ during the same session the sample used for testing was collected. As we can observe, the highest prediction accuracy for all three emotional states was obtained when the DSC was used for both training and testing. In that case, we obtained 89.3% accuracy for depression, 79.2% accuracy for anxiety, and 92.1% for stress. If we kept the DSC for training and we used nonemotion-elicited samples from the DSR for testing the architecture, we observed the accuracy, sensitivity, and specificity decrease at less than 2%, which shows that, when training and testing using samples from the same subject, inducing emotions was essential only for training the system, while for testing we could use video recordings collected in naturalistic conditions. This observation makes this architecture practical, as it only needs to be pretrained with emotion-induced samples from the subject, while subsequent assessments of a subject’s DASS levels can be done in real conditions, without inducing emotions, and hence it can be used in real time to monitor the facial dynamics of a patient and evaluate the level of stress, anxiety, or depression. We observe in Table 1 and Figure 10 that the highest accuracy for all three emotional states was obtained for Normal (90.4% for depression in the most practical case when we trained using samples from the DSC and tested using samples from the DSR, 84.1% for anxiety and 94.2% for stress) and Extremely Severe levels (93.2% for depression, 82.9% for anxiety, and 93.5% for stress). For the other intermediary levels, the accuracy was lower, and these were most often mistaken for neighboring levels (e.g., the Mild level was mostly mistaken for the Moderate level for depression and stress, and for the Normal level for anxiety). We conclude that the proposed architecture could successfully discriminate between depressive/nondepressive, anxious/nonanxious, and stressed/nonstressed subjects, but for discriminating between DASS levels, the results were lower, and other methods need to be researched to enhance this further, such as including more AUs in the facial analysis, fusing the FFNN-based method with other state-of-the-art methods for facial analysis, or using the face modality alongside other modalities (such as voice) in a multimodal approach.

**Figure 11:**
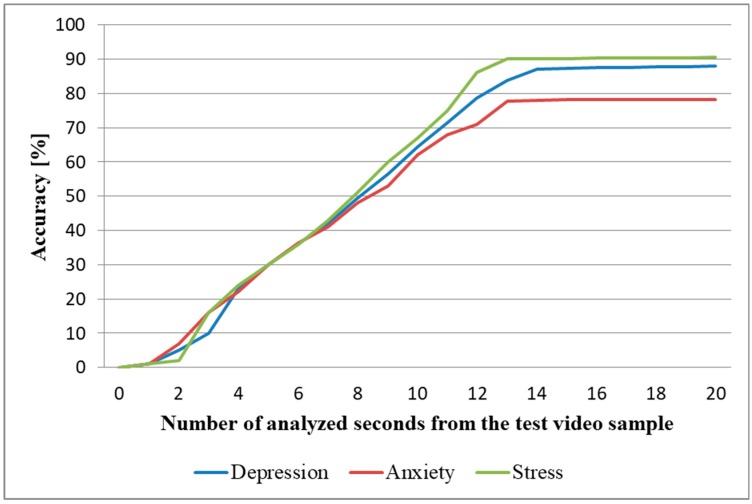
Prediction accuracy based on the number of seconds analyzed from the test video samples (intrasubject methodology).

In the most practical case, when we trained the system using samples from the DSC and we tested it using samples from the DSR, the average time needed to predict the three emotional states was 14 s (as seen in Figure 11). As processing one second of video recording lasts, on average, three seconds, the time needed for predicting the DASS levels in the intrasubject methodology was 56 s, significantly faster than the time needed to complete the SAQ (25.4 min), which makes this architecture fast enough to be used in real time and attractive for health practitioners to evaluate and monitor the DASS levels of their patients.

**Figure 12:**
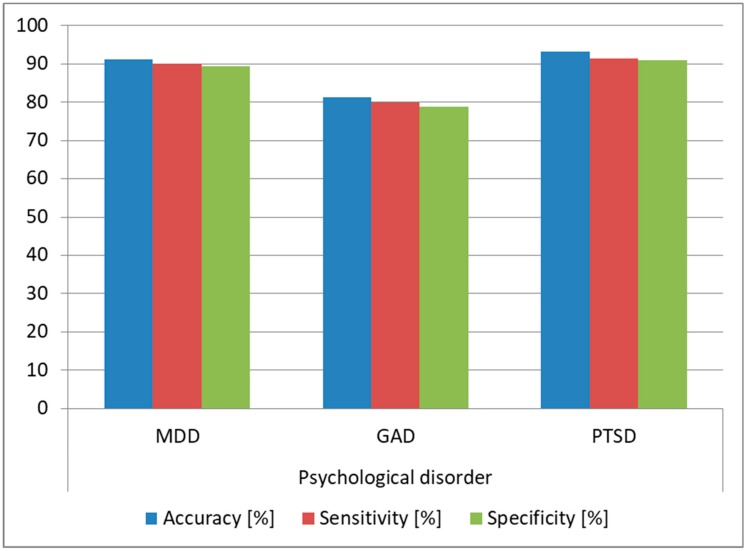
Prediction accuracy for major depressive disorder (MDD), generalized anxiety disorder (GAD), and post-traumatic stress disorder (PTSD) (intrasubject methodology).

As we observed that the architecture provided high accuracy for predicting Normal and Extremely Severe levels, we evaluated the same architecture on the task of determining if the analyzed subjects were affected by MDD, GAD, or PTSD. We trained the architecture using the DSC samples from the subjects affected by these conditions and we tested the architecture on the samples from the DSR (since this was the most practical case). We also determined the optimal FFNN hyperparameters in this case, keeping the same 80:20 split ratio, and we obtained as optimal an FFNN with two hidden layers, tanh as the activation function for the first hidden layer and sigmoid for the second hidden layer and the output layer. The structure was similar to the one obtained for predicting DASS levels, the difference being in the number of neurons for the two hidden layers: in this case, we needed 30 neurons for the first hidden layer and 25 for the second hidden layer. During training, the bias for the hidden layers was also set to 0. The FFNN had three output neurons providing a binary result for each of the three disorders (MDD, GAD, and PTSD), such that if the subject was suffering from the disorder, the output was 1, and if he/she was healthy, the output was 0. The results are displayed in Figure 12, and we can see that we obtained over 90% prediction accuracy and over 89% sensitivity and specificity for predicting MDD and PTSD, which shows that our architecture can be successfully used for diagnosing these disorders.

### 3.3. Intersubject Methodology

In the intersubject methodology, we trained the architecture using samples pertaining to a set of subjects and we tested it on samples associated with a different subject that was not used for training, varying the testing and training datasets (DSC, DSR, or both). We used a leave-one-out approach such that we trained on DSC, DSR, or both DSC and DSR samples from 127 subjects, and we tested the samples from the remaining subject. We repeated the tests until all samples were used for testing the system and all subjects had been involved in the testing phase, and we averaged the results, which can be observed in Table 2.

The results were consistent with those obtained in the intrasubject methodology, with the highest prediction accuracy being obtained when we trained and tested the architecture using samples from the DSC, in this case reaching 79.8% accuracy for depression, 68.7% accuracy for anxiety, and 81.5% accuracy for stress. We observed that when we used samples from the DSR for both testing and training, the accuracy was 14% lower, while when we trained the system using samples from the DSC from 127 subjects and tested it with samples from the DSR from the remaining subject, the accuracy was only 1.5% lower.

We reached a conclusion similar to that from the intrasubject methodology, with the emotion-inducing facial activity adding more value only in the training phase, while for testing we could use recordings collected in nonemotion eliciting scenarios without any significant influence on the accuracy, sensitivity, and specificity of the architecture. For the intrasubject methodology, this observation was even more valuable, as the system could be pretrained using video recordings of subjects whose emotions were induced, while the end-user could assess the DASS levels in naturalistic scenarios, without the need for training the system with his/her own samples.

**Figure 13:**
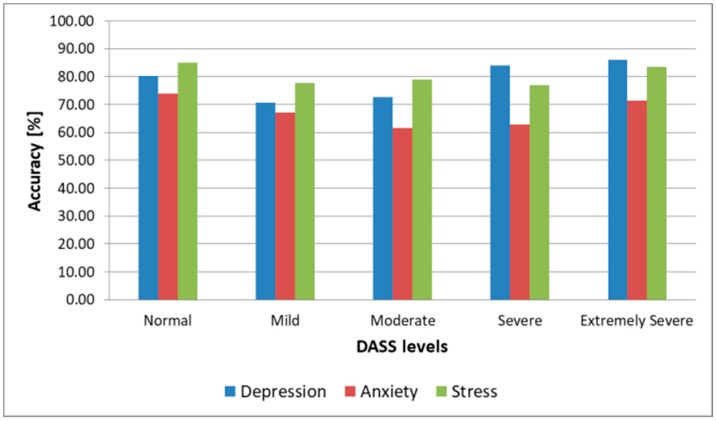
DASS levels prediction accuracy (intersubject methodology).

We also observed (Figure 13) that the architecture offered an accuracy of over 80% for stress and depression and over 71% for anxiety when predicting Normal and Extremely Severe levels, confirming, in this case as well, the fact that it can successfully discriminate between healthy subjects and those affected by depression, anxiety, and stress, but cannot discriminate with the same accuracy between intermediary levels (Mild, Moderate, and Severe), with these being often mistaken for their neighboring levels (e.g., the Mild level mistaken for the Normal level for depression and for the Moderate level for anxiety and stress), similar to the intrasubject methodology results. The fact that the results obtained with the two methodologies were similar shows that the proposed architecture is robust.

**Figure 14:**
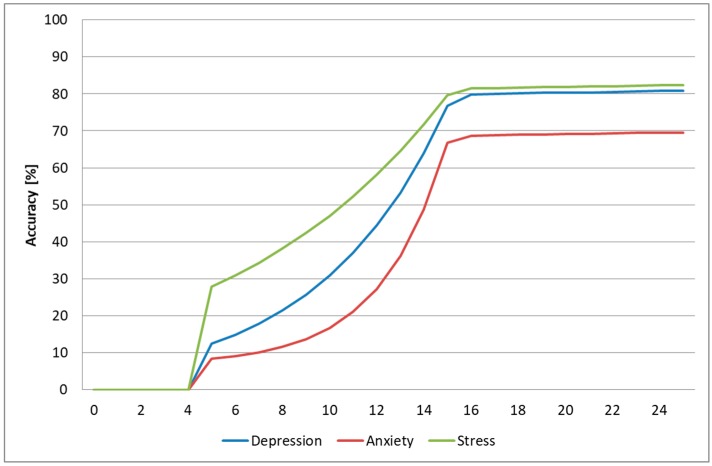
Prediction accuracy based on the number of seconds analyzed from the test video samples (intersubject methodology).

The average duration of the video sample needed for predicting all three emotional states was 16 s (as seen in Figure 14), and therefore the average DASS level prediction time was 64 s, a lot faster than the time needed for completing the SAQ, and hence the architecture can be used for monitoring the three emotional states in real time and can be an attractive tool for health practitioners to assess DASS levels, if the prediction of intermediary DASS levels is improved.

**Figure 15:**
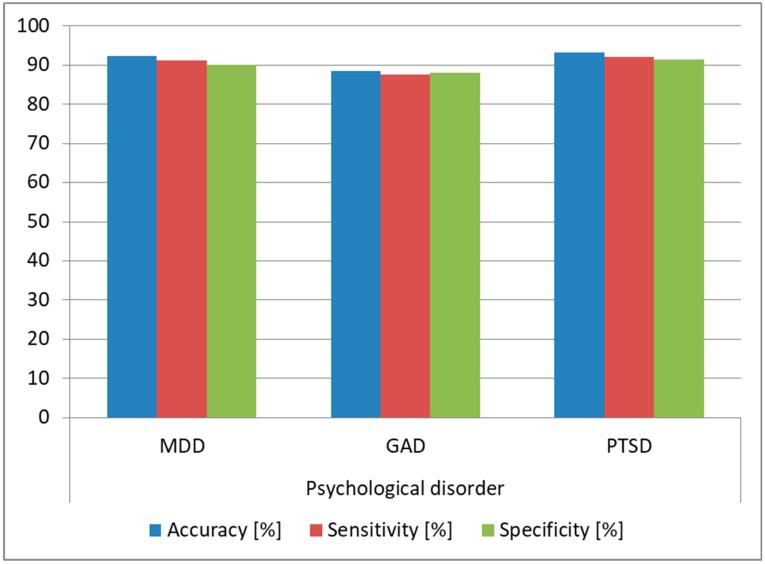
Prediction accuracy for MDD, GAD, and PTSD (intersubject methodology).

We performed a test similar to the one conducted for the intrasubject methodology, and we evaluated the architecture on the task of determining if the analyzed subjects were affected by MDD, GAD, or PTSD. We used the same FFNN and hyperparameters detailed for the intrasubject methodology. We trained the architecture using DSC samples from 127 subjects and we tested it using samples from the DSR for the remaining subject, repeating the tests until all samples and all subjects were involved in the testing phase. The results are displayed in Figure 15, and we observed over 93% accuracy for MDD and PTSD and over 85% accuracy for GAD, which shows that the architecture can be used as a tool for diagnosing these psychological disorders. Moreover, the accuracy obtained in the intersubject methodology was higher than the one obtained in the intrasubject methodology, which is explained by the fact that the training data variability was higher in the intersubject tests.

**Figure 16:**
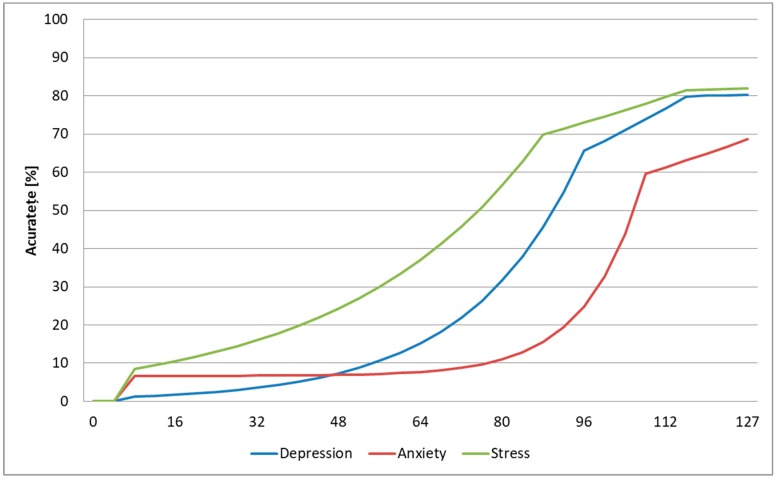
Prediction accuracy based on the number of subjects used for training (intersubject methodology).

We also tested the accuracy of the architecture based on the number of subjects we used for training in order to determine the lowest number of subjects required to get satisfying results. For this, we trained the system on DSC samples from a set of subjects and tested it on DSR samples from subjects that were not involved in training, repeating the tests until all DSR samples and all users were used for testing and averaging the results. The results are displayed in Figure 16, and we show that we needed a minimum of 116 subjects for stress and depression for the system to provide reasonable results, while for anxiety the 127 subjects used in this study did not seem to be sufficient. This also shows that, for stress and depression, increasing the number of subjects in the database might not improve significantly the prediction accuracy, and hence other state-of-the-art methods can be fused with the FFNN-based method presented in this study to improve the accuracy more. For anxiety, increasing the number of subjects used for training could definitely lead to an increase in the prediction accuracy of this emotional state.

### 3.4. Correlations between Emotions, FACS, and DASS Levels

As we observed that the samples collected when emotion was induced led to higher accuracy when used in the training phase, we built a background application that searched for AUs of intensity D or E in the FM whenever an Extremely Severe level was correctly predicted. The top four AUs determined for each of the three emotional states are displayed in Table 3. We see that these AU combinations were unique for each of the three emotional states, and we modified the architecture such that when the AUs were present at intensity level D or E, we provided the Extremely Severe score without passing through the neural network structure. This enhancement led to increasing the accuracy for predicting the Extremely Severe and Severe levels by 8% (as the Severe levels were less often mistaken for Extremely Severe thanks to this enhancement) and the overall accuracy of the architecture by 5%.

We also performed intersubject tests in which we trained the FDASSNN with samples from the DSC pertaining to one emotion only, from 127 subjects, and we tested it on samples from the DSR pertaining to the remaining subject, repeating the tests until all samples from the DSR and all subjects were involved in the testing phase, as well as until all emotions were used for the training phase. We identified that when using the samples where happiness or sadness were induced for training, all depression levels could be predicted with over 85% accuracy; when using samples where surprise or disgust were induced for training, the anxiety levels were predicted with over 80% accuracy; and when using the samples where sadness and disgust were induced for training, all of the stress levels were predicted with over 90% accuracy. The overall accuracy obtained in these cases and the associated emotions are presented in Table 4. Therefore, we show that by selectively training the architecture with samples where specific emotions were induced, not only did the accuracy improve, but intermediary levels were predicted with higher accuracy, too.

### 3.5. Comparison to State-of-the-Art Methods

In order to compare the performance of the proposed architecture to that of the state-of-the-art methods, we used the database proposed in this study as well as ANUStressDB [12] for evaluating stress and AVEC2014 [19] for evaluating depression. We compared our proposed method to the state-of-the-art systems presented in the introductory section as well as to several classic backbone networks, such as VGG (VGG-11, VGG-13, VGG-16, VGG19), ResNet (ResNet-18, ResNet34, ResNet-50, ResNet-101, ResNet-152), Inception (v3 and v4), GoogLeNet, BN-Inception, and AlexNet [78].

**Figure 17:**
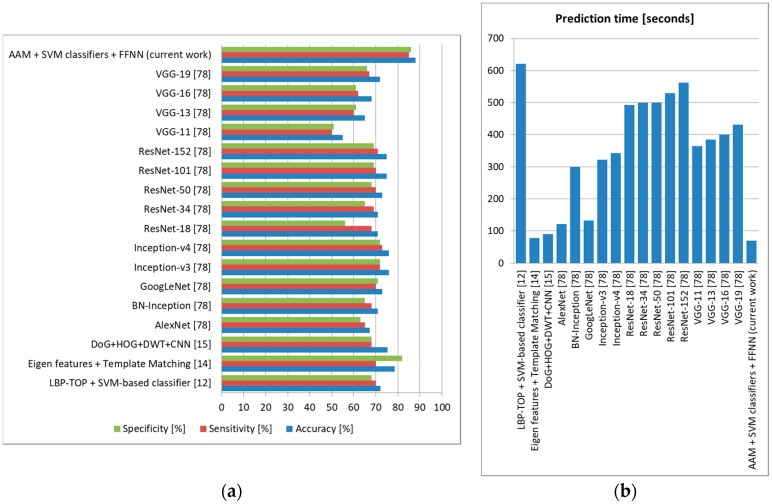
Stress prediction comparison to state-of-the-art methods (ANUStressDB [12]). (**a**) Average accuracy, sensitivity, and specificity, (**b**) Average prediction time (seconds).

For stress, we kept the multiclass SVM classifiers trained using CK+ and we trained the FFNN based on ANUStressDB [12], using leave-one-out cross-validation and evaluating only the results provided by the output neuron assessing stress levels (for Normal and Mild, we considered the subject to be nonstressed, while for other levels, we considered the subject to be stressed). The results are displayed in Figure 17. As we can observe, our architecture surpassed all other state-of-the-art methods, offering 88% accuracy with 85% sensitivity and 86% specificity in the task of discriminating between stressed and nonstressed subjects, with a lowest prediction time of 70 s.

**Figure 18:**
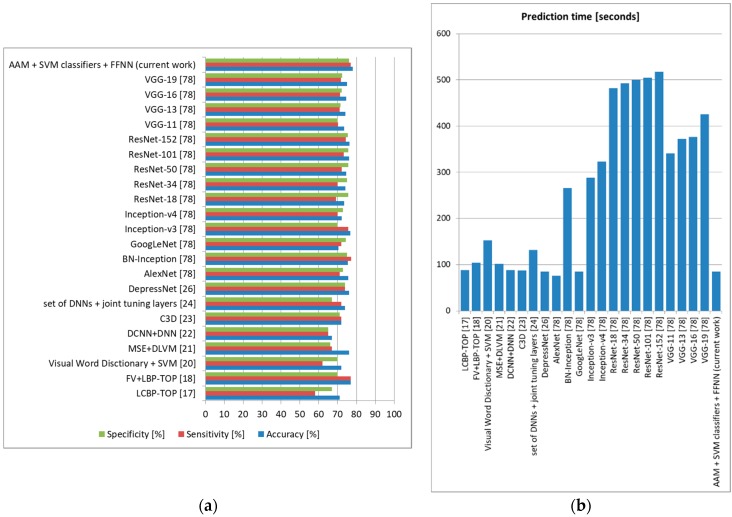
Depression prediction comparison to state-of-the-art methods (AVEC2014 [19]). (**a**) Average accuracy, sensitivity, and specificity, (**b**) Average prediction time (seconds).

For depression, we used AVEC2014 [19], and the results are detailed in Figure 18. As can be observed, our architecture offered accuracy 1.5% higher than the FV LBP-TOPs method [18], and it also offered one of the lowest prediction times (85 s) (AlexNet offered the lowest prediction time of 76 s in this case, but with significantly lower accuracy).

**Figure 19:**
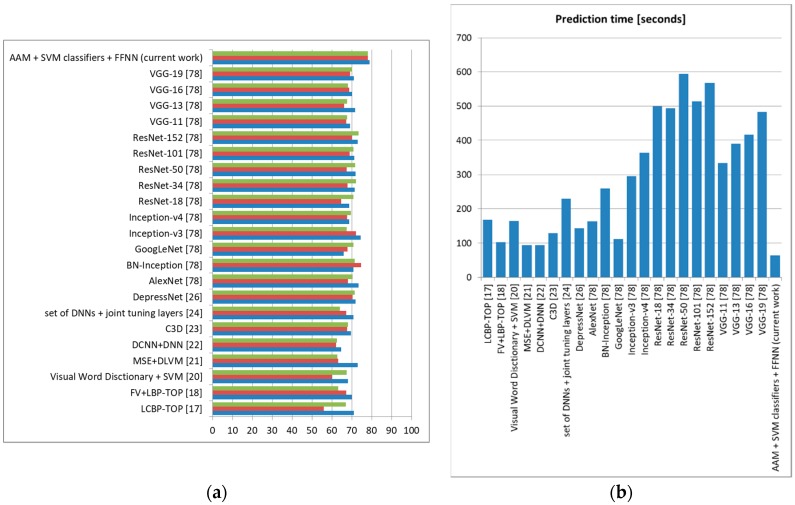
Depression prediction comparison to state-of-the-art methods (own database). (**a**) Average accuracy, sensitivity, and specificity, (**b**) Average prediction time (seconds).

**Figure 20:**
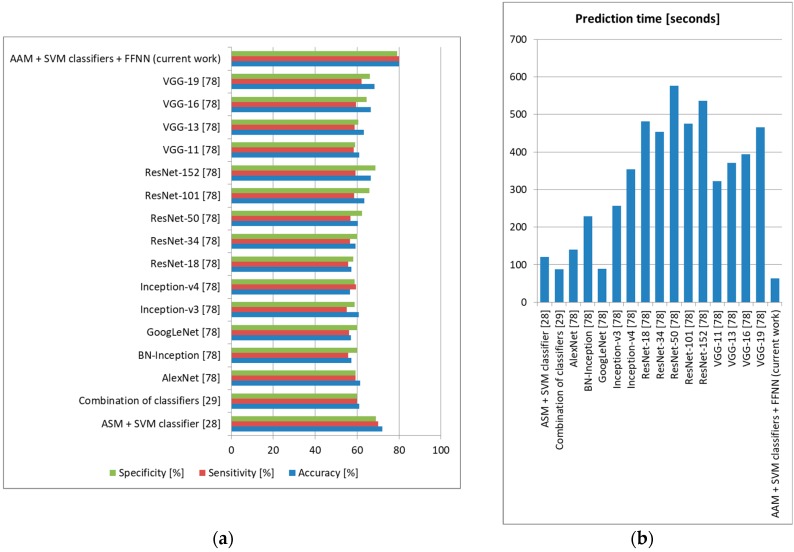
Anxiety prediction comparison to state-of-the-art methods (own database). (**a**) Average accuracy, sensitivity, and specificity, (**b**) Average prediction time (seconds).

**Figure 21:**
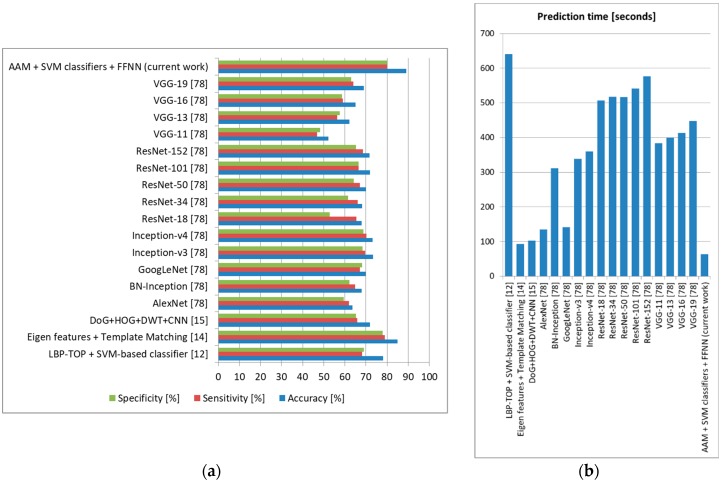
Stress prediction comparison to state-of-the-art methods (own database). (**a**) Average accuracy, sensitivity, and specificity, (**b**) Average prediction time (seconds).

We also tested these state-of-the-art methods on our own database using the intersubject methodology with a leave-one-out approach (training the system on DSC samples from 127 subjects and testing it on DSR samples from the remaining subject, repeating the tests until all DSR samples and all subjects were used for testing and averaging the results), and the accuracy, sensitivity, and specificity obtained can be observed in Figure 19 (depression), Figure 20 (anxiety), and Figure 21 (stress). 

For depression, the proposed architecture offered accuracy up to 5% higher compared to other methods in the literature, surpassing these methods also in terms of sensitivity and specificity. For anxiety, we obtained 5% higher accuracy than the method employing ASM and SVM classification [28], as well as sensitivity and specificity up to 4% higher. For stress, our architecture offered 3% higher accuracy and 2% higher sensitivity and specificity compared to the most accurate method in the literature (using Eigen features and template matching) [14]. In all these tests, the method proposed in this study offered the lowest prediction time, which shows that it is robust and can be confidently used for real-time monitoring. Because our architecture offered an accuracy higher than other systems that evaluate these three emotional states separately, we can state that the higher results were not only as a result of the use of FACS and an optimal FFNN, but also because we treated the three emotional states together, incorporating their possible correlations [1].

## 4. Discussion

We proposed a novel multilayer neural network-based architecture for determining DASS levels by analyzing the facial expression dynamics of a subject using FACS. This is the first study of this kind in the literature that has proposed a unique architecture built on three layers designed to offer high accuracy and low prediction time: in the first layer, the video frames are normalized, and the analyzed AUs are classified using an AAM-based method and a set of multiclass SVMs; in the second layer, a matrix is built containing the intensity levels of the selected AUs for each video frame; in the third layer, we determine the optimal structure of an FFNN trained to analyze the patterns from the matrix computed in the second layer and predict the DASS levels (Normal, Mild, Moderate, Severe, or Extremely Severe) for each of the three emotional states. To test the proposed architecture, we built a new database containing frontal face recordings of subjects as well as SAQ results in both controlled scenarios (when emotion was induced in the subjects) and random scenarios (when no emotion was induced), ensuring it displayed both intrasubject and intersubject variability. We obtained an accuracy of 87.2% for depression, 77.9% for anxiety, and 90.2% for stress with the intrasubject methodology and 78.6% for depression, 67.3% for anxiety, and 80.4% for stress with the intersubject methodology. In both methodologies, we showed that the intermediary DASS levels (Mild, Moderate, and Severe) were predicted with an accuracy significantly lower, as these levels were most often mistaken for neighboring levels, and therefore the proposed architecture can successfully predict whether a subject is affected by depression, anxiety, or stress or is healthy, but does not offer the same accurate results for distinguishing between the severity levels of the three emotional states. Other methods can be fused with the current FFNN-based one or a multimodal approach can be evaluated to increase the accuracy of the intermediary level prediction. The average prediction time was 56 s (intrasubject methodology) and 64 s (intersubject methodology), faster than the time needed to complete the SAQ (25.4 min), and therefore the architecture is fast and can be used in real time to monitor the three emotional states, an attractive alternative to questionnaire-based methods and a tool to help health practitioners if the accuracy for predicting the intermediary DASS levels is improved. 

We used the proposed architecture to discriminate between healthy subjects and those affected by major depressive disorder (MDD), post-traumatic stress disorder (PTSD), or generalized anxiety disorder (GAD), and we obtained over 93% accuracy in predicting MDD and PTSD and over 85% accuracy in predicting GAD: hence, the architecture can be used by health practitioners as a tool for diagnosing these psychological disorders. We determined a set of connections between DASS levels and analyzed AUs, with this being the first study in the literature that has identified these connections. By modifying the architecture to include these connections, the overall accuracy increased up to 5%. We also identified correlations between the induced emotions and DASS levels, and we showed that by inducing only specific emotions for each of the three emotional states, the accuracy improved up to 8%, and the intermediary levels were also predicted with high accuracy. This shows that inducing specific emotions triggered a set of facial features that could be more relevant for each emotional state analyzed. 

We compared our proposed architecture to other state-of-the-art methods, and we showed that it provided up to 5% more accuracy for depression and anxiety prediction and 3% higher accuracy for stress prediction. 

The architecture can be further enhanced by analyzing other AUs or fusing at the feature or score level other methods with the FFNN-based one proposed in this study. We also showed that increasing the number of subjects and samples in the database could lead to increasing the prediction accuracy for anxiety. Since this architecture was designed to analyze exclusively frontal face video recordings, other methods (such as using facial landmarks) to increase accuracy in the case of head pose changes or partial face occlusions can be explored to enhance the proposed architecture. The architecture can be used as a diagnostic tool for health practitioners, but can also be integrated in applications such as virtual psychology [3], computer-driven therapy [3], career counseling [3], or even diagnoses of physical diseases based on patterns of stress, anxiety, or depression levels [4,5,6].

## Figures and Tables

**Table 1:** DASS levels prediction accuracy, sensitivity, specificity, and average duration of analyzed video sample in intrasubject methodology.

DSTR		DSC			DSC			DSR			DSR			DSC + DSR			LMM
DSTS		DSC			DSR			DSR			DSC			DSC + DSR			
MM		AC	SE	SP	AC	SE	SP	AC	SE	SP	AC	SE	SP	AC	SE	SP	
Depression	(1)	91.1	91	91	90.4	89.7	89.9	82.1	81.6	81.8	85.2	84.4	84.9	86.3	85.7	86	(2)
	(2)	80.2	79.9	79.9	78.5	78	78	71.9	71.7	71.6	74.6	73.7	74.4	76	75.5	75.7	(3)
	(3)	82.1	81.1	81.7	81.1	80.7	80.8	72.7	71.8	72.3	75.9	75.3	75.5	77	76.8	76.6	(1)
	(4)	94	93.9	93.8	92.8	91.9	92.6	84.9	84	84.7	87.5	86.8	87	88.6	87.9	88.1	(3)
	(5)	95.2	93.9	94.3	93.2	92.5	92.8	86.1	85.8	85.6	90.3	90.1	90	91.7	91.2	91.3	(4)
	Avg.	89.3	88.1	88.3	87.2	86.6	86.9	79.6	78.9	79.1	82.7	82.4	82.3	83.9	83.1	83.6	
ADVS		12			12			17			16			14			
Anxiety	(1)	85.1	84.8	84.7	84.1	83.5	83.7	74.9	74.6	74.7	78.3	77.3	77.8	79.5	79.2	79.1	(2)
	(2)	78	78	78.2	77.2	77	76.7	67.9	67.5	67.7	71.7	70.9	71.3	72.9	72.7	72.6	(1)
	(3)	72.2	71.4	71.6	70.8	70.6	70.3	63	62.1	62.8	66	65.8	65.6	67.1	66.4	66.9	(4)
	(4)	76.1	74.9	75.4	74.7	73.9	74.3	64.1	63.2	63.8	67.1	66.6	66.9	68.4	68.1	68.1	(5)
	(5)	84.1	83.7	83.8	82.9	82.5	82.4	72.8	72.1	72.5	75.5	75.3	75.2	76.9	75.9	76.6	(4)
	Avg.	79.2	78.4	78.6	77.9	77	77.7	68.6	67.9	68.3	71.7	71.5	71.4	73	72.2	72.6	
ADVS		14			14			19			17			16			
Stress	(1)	95.1	94.9	94.9	94.2	93.2	93.8	87.1	86.2	86.7	89.5	88.7	89.1	90.5	90.2	90.1	(2)
	(2)	89.2	88.8	88.9	88	87.8	87.6	77.7	76.8	77.5	81	80.5	80.6	82.4	81.5	82	(3)
	(3)	89.1	88.4	88.9	88.3	87.6	88.1	80.8	80.3	80.5	82.9	82	82.5	84.1	83.8	83.9	(2)
	(4)	89.2	87.9	88.3	87.1	86.1	86.8	77.4	76.5	77.1	81.7	81.1	81.5	83	82.1	82.8	(5)
	(5)	95	94	94.5	93.5	93.1	93.3	83.9	83.2	83.6	88.2	87.7	88	89.6	88.6	89.1	(4)
	Avg.	92.1	90.9	91.1	90.2	89.7	89.8	81.4	81.2	81.1	84.7	83.8	84.3	85.9	85.7	85.7	
ADVS		14			14			19			17			16			

AC: accuracy; SE: sensitivity; SP: specificity; Avg: average; (1): normal level; (2): mild level; (3): moderate level; (4): severe level; (5): extremely severe level; DSTR: dataset training; DSTS: dataset testing; ADVS: average duration of analyzed video sample (seconds); MM: measure method; LMM: DASS level most mistaken for.

**Table 2:** DASS levels prediction accuracy, sensitivity, specificity, and average duration of analyzed video samples in intersubject methodology.

DSTR		DSC			DSC			DSR			DSR			DSC + DSR			LMM
DSTE		DSC			DSR			DSR			DSC			DSC + DSR			
MM		AC	SE	SP	AC	SE	SP	AC	SE	SP	AC	SE	SP	AC	SE	SP	
Depression	(1)	81.2	78.0	79.8	80.1	76.9	78.7	68.1	65.4	67.0	67.3	65.9	65.6	72.2	70.0	70.5	(2)
	(2)	71.9	70.6	70.1	70.6	68.3	68.8	52.9	50.0	51.0	53.1	50.1	51.3	57.1	55.9	55.4	(1)
	(3)	73.8	71.3	72.1	72.6	71.4	71.3	55.5	52.2	53.6	55.4	53.5	54.3	60.3	57.8	58.8	(2)
	(4)	85.1	82.7	83.2	83.9	82.3	82.9	73.8	70.2	72.7	73.1	70.4	71.1	77.7	74.8	75.9	(3)
	(5)	87.1	83.2	85.2	85.9	84.5	84.4	75.0	73.7	73.9	75.7	72.6	74.7	79.8	77.6	78.6	(4)
	Avg.	79.8	77.2	78.1	78.6	76.7	77.2	65.1	62.3	63.6	64.9	62.5	63.4	69.4	67.2	67.8	
ADVS		11			16			18			14			13			
Anxiety	(1)	75.2	72.0	73.7	74.0	71.2	72.1	65.6	61.6	63.8	64.9	61.1	63.0	69.4	67.8	68.1	(2)
	(2)	68.5	65.5	67.3	67.0	64.6	65.7	50.3	49.2	49.0	49.9	46.2	48.4	54.8	52.6	52.8	(3)
	(3)	63.0	60.9	61.4	61.6	58.8	60.5	53.0	51.8	51.2	53.7	49.9	52.6	58.4	55.5	57.3	(2)
	(4)	64.2	63.1	62.3	62.7	60.8	60.9	50.7	49.4	49.3	50.3	47.0	49.1	54.6	51.3	53.3	(3)
	(5)	72.4	70.7	71.4	71.3	69.0	69.4	55.1	52.2	53.2	55.1	52.4	54.0	60.1	57.4	58.4	(4)
	Avg.	68.7	66.4	67.2	67.3	64.9	65.7	54.9	52.8	53.3	54.8	51.3	53.4	59.5	56.9	58.0	
ADVS		12			16			20			14			14			
Stress	(1)	86.0	83.2	84.8	85.0	81.7	83.5	74.1	70.8	72.2	74.4	71.7	72.8	79.4	78.3	77.5	(2)
	(2)	78.8	76.5	76.8	77.6	73.6	75.9	60.4	56.4	59.0	60.8	58.2	59.6	65.4	64.1	63.8	(3)
	(3)	80.0	77.9	78.1	78.8	75.0	77.6	71.0	69.9	69.0	70.8	69.2	69.2	75.1	72.4	73.4	(4)
	(4)	78.2	76.5	76.3	77.0	75.1	75.3	69.0	66.7	67.6	68.6	65.8	67.1	73.0	70.5	71.6	(3)
	(5)	84.5	81.3	82.9	83.5	82.2	81.6	75.4	73.8	74.0	75.1	71.2	73.4	79.9	78.2	78.4	(4)
	Avg.	81.5	79.1	79.8	80.4	77.5	78.7	70.0	67.5	68.4	70.0	67.2	68.4	74.6	72.7	73.0	
ADVS		12			15			18			14			14			

AC: accuracy; SE: sensitivity; SP: specificity; Avg.: average; (1): normal level; (2): mild level; (3): moderate level; (4): severe level; (5): extremely severe level; DSTR: dataset training; DSTS: dataset testing; DAVS: average duration of analyzed video sample (seconds); MM: measure method; LMM: DASS level most mistaken for.

**Table 3:** Correlations between FACS and DASS levels.

Emotional State	AU (Intensity Level D or E)	Accuracy (%)
Depression	AU6, AU12, AU15, AU26	84.51
Anxiety	AU2, AU9, AU25, AU45	72.92
Stress	AU1, AU6, AU12, AU15	88.41

**Table 4:** Correlations between emotions and DASS levels.

Emotional State	Induced Emotion	Accuracy (%)
Depression	Happiness, sadness	87.71
Anxiety	Surprise, disgust	82.13
Stress	Sadness, disgust	93.21
